# Nurse Practitioner’s Role as 3D Team Leader

**DOI:** 10.1093/geroni/igaa057.2679

**Published:** 2020-12-16

**Authors:** Kathleen Obuchon

**Affiliations:** UConn Health, Farmington, Connecticut, United States

## Abstract

The nurse practitioner’s (NP) clinical activities during the 12-month intervention period include 4 monthly in-home visits and 8 monthly telephone contacts. This presentation will detail the clinical assessments and activities conducted during the initial home visit, and how subsequent home visit activities and interventions are structured for older adults and their informal caregivers depending on whether older adults have dementia, depression, and/or recent delirium. Because the potential for medication-related problems is a critical concern for older adults with cognitive vulnerability, this presentation also will detail how the NP works with the 3D Team pharmacist to determine potential inappropriate medications through a review and reconciliation process, and how the NP and pharmacist summarize these results and correspond accordingly with the older adult’s primary care physician. Finally, this presentation will explain how the NP manages communication among members of the 3D Team who provide interventions to the same older adult and caregiver.

